# Homogenous generation of dopaminergic neurons from multiple hiPSC lines by transient expression of transcription factors

**DOI:** 10.1038/s41419-019-2133-9

**Published:** 2019-11-27

**Authors:** Sameehan Mahajani, Anupam Raina, Claudia Fokken, Sebastian Kügler, Mathias Bähr

**Affiliations:** 1 Department of Neurology, University Medical Center Göttingen, Göttingen, Germany; 2 Center for Nanoscale Microscopy and Molecular Physiology of the Brain at Department of Neurology, University Medical Center Göttingen, Göttingen, Germany

**Keywords:** Induced pluripotent stem cells, Stem-cell differentiation, Parkinson's disease

## Abstract

A major hallmark of Parkinson's disease is loss of dopaminergic neurons in the substantia nigra pars compacta (SNpc). The pathophysiological mechanisms causing this relatively selective neurodegeneration are poorly understood, and thus experimental systems allowing to study dopaminergic neuron dysfunction are needed. Induced pluripotent stem cells (iPSCs) differentiated toward a dopaminergic neuronal phenotype offer a valuable source to generate human dopaminergic neurons. However, currently available protocols result in a highly variable yield of dopaminergic neurons depending on the source of hiPSCs. We have now developed a protocol based on HBA promoter-driven transient expression of transcription factors by means of adeno-associated viral (AAV) vectors, that allowed to generate very consistent numbers of dopaminergic neurons from four different human iPSC lines. We also demonstrate that AAV vectors expressing reporter genes from a neuron-specific hSyn1 promoter can serve as surrogate markers for maturation of hiPSC-derived dopaminergic neurons. Dopaminergic neurons differentiated by transcription factor expression showed aggravated neurodegeneration through α-synuclein overexpression, but were not sensitive to γ-synuclein overexpression, suggesting that these neurons are well suited to study neurodegeneration in the context of Parkinson’s disease.

## Introduction

Parkinson’s disease is one of the most common neurological disorders. The disease is characterized by loss of dopaminergic neurons in the substantia nigra pars compacta. Various studies have shown that aggregation of an abnormally folded protein, α-synuclein, results in intracellular inclusions within cell bodies and processes of these neurons called Lewy bodies^1–3^. Besides mutations, expression levels of α-synuclein influence viability of dopaminergic neurons^4,5^. We have previously shown that overexpression not only of α-synuclein^6^ and β-synuclein is toxic to rat primary cortical neurons in vitro, but also to dopaminergic neurons in vivo^7^. In order to move on and decipher the molecular pathology that leads to dopaminergic neurodegeneration, human cell-based model systems are essential and differentiation of human induced pluripotent stem cells (hiPSCs) to dopaminergic neurons has been suggested to provide such a model system.

Dopaminergic neurons can be generated from hiPSCs using various published protocols^8–12^. One of the most efficient protocol for the generation of dopaminergic neurons uses a combination of several pharmacological compounds^13^. This protocol has been successfully exploited, although with highly varying amounts of dopaminergic neurons being generated, ranging from 8 to 85% of the final cell number^14–19^. The variation in the numbers of differentiated dopaminergic neurons among these publications is likely due to the differences in hiPSC lines and their handling among several labs. In addition, however, the strategy itself might not be optimal and we therefore reasoned that expression of transcription factors related to dopaminergic neuronal differentiation might be able to generate a better reproducible and more homogenous outcome concerning numbers and cell type from several different hiPSC lines.

In this study, we report that the protocol using pharmacological compounds indeed generated variable numbers of neurons in general, and percentages of dopaminergic neurons specifically, from four different hiPSC lines. These results suggest that these compounds trigger divergent cellular activation states in different cell lines and thus might not be able to pattern different hiPSC lines with sufficient stringency. In contrast, exploiting AAVs to express rat transcription factors (rTFs) rLmx1a, rNurr1 or rPitx3 in undifferentiated hiPSCs generated from four different individuals, showed much more consistent results. The overexpression of any one of these three transcription factors is sufficient to generate similar numbers of dopaminergic neurons from four different hiPSC lines. Intriguingly, the HBA promoter driven expression of these rTFs is transient in nature, i.e. self-limiting its expression by 10 days post transduction (DPT). We also demonstrate that rLmx1a acts as a master regulator and controls the patterning of hiPSCs into dopaminergic neurons. Moreover, we report that a viral vector expressing a reporter gene under the control of neuron-specific hSyn1 promoter can be used as a novel surrogate marker to monitor neuronal maturation. Finally, we demonstrate that our transcription factor-differentiated neurons can be readily used to study neurotoxicity of α-synuclein.

## Results

### Significant variation in generation of dopaminergic neurons using pharmacological compounds from different hiPSC lines

**Fig. 1:**
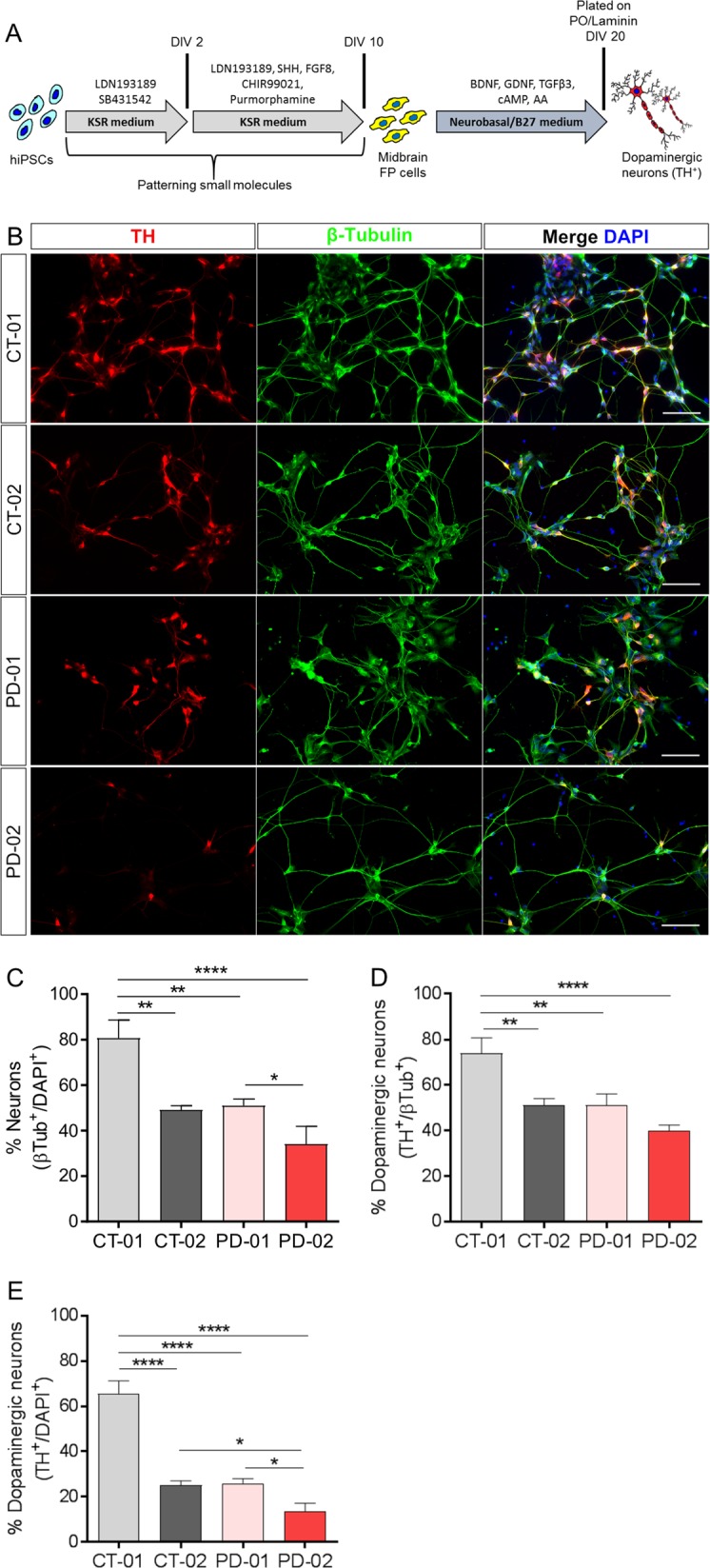
Quantification of the percentages of neurons and dopaminergic neurons obtained from different hiPSC lines using pharmacological compounds. **a** Schematic representation of the use of pharmacological compounds to pattern hiPSCs to generate TH^+^ dopaminergic neurons at DIV 25. The patterning protocol consists of inhibiting SMAD signaling and activating SHH and WNT signaling to obtain midbrain floor-plate precursors by DIV 11. These cells in the presence of growth factors mature into TH^+^ dopaminergic neurons. **b** Representative fluorescence images of immunoreactivity for the dopaminergic neuronal marker TH (red) and the pan-neuronal marker β-Tubulin (green) at DIV 25. Nuclei are counterstained with DAPI (blue). Scale bars: 100 µm. **c** Quantitative analysis of the percentage of neurons obtained after patterning with pharmacological compounds. Data represent the average percentage of neurons of total cell numbers in culture (β-Tubulin^+^/DAPI^+^). *p < 0.05; **p < 0.01; ****p < 0.0001, one-way ANOVA followed by Bonferroni’s post-hoc test. **d** Quantitative analysis of total percentage of dopaminergic neurons obtained after patterning with pharmacological compounds. Data represent the average percentage of total dopaminergic neurons out of all neurons in culture (TH^+^/β-Tubulin^+^). **p < 0.01; ***p < 0.001, one-way ANOVA followed by Bonferroni’s post-hoc test. **e** Quantitative analysis of total percentage of dopaminergic neurons obtained after patterning with pharmacological compounds. Data represent the average percentage of total dopaminergic neurons out of all cells in culture (TH^+^/DAPI^+^). *p < 0.05; ****p < 0.0001, one-way ANOVA followed by Bonferroni’s post-hoc test. In all graphs, bars represent the average percentage ± SD from at least three independent differentiations for each of the four hiPSC lines.

To investigate the efficacy of pharmacological compounds to generate dopaminergic neurons from four different hiPSC lines, we used the protocol published^13^, where SMAD signaling is inhibited (using LDN/SB) and SHH and WNT signaling is activated (using SHH/PUR and CHIR, respectively). In this patterning protocol, the specific factors are applied over 20 days (Fig. 1a). We analyzed differentiated cells by immunostaining for the neuron-specific marker β-Tubulin (Fig. 1b; green) and the dopaminergic neuronal marker tyrosine hydroxylase (TH) (Fig.1b; red). This was done with two hiPSC lines obtained from control individuals (CT-01 and CT-02) and with two lines obtained from idiopathic Parkinson’s Disease (PD) patients (PD-01 and PD-02). Upon quantification of three independent differentiations for each hiPSC line, we observed that the number of β-Tubulin^+^ neurons generated after patterning was significantly different in these hiPSC lines (Fig. 1c). The pharmacological patterning protocol generated highly variable percentages of neurons from CT-01 (~81%), CT-02 (~50%), PD-01 (~50%) and PD-02 (~34%). Similarly, the number of dopaminergic neurons generated from these four hiPSC lines were also significantly different (Fig. 1d, e). The highest numbers of differentiated dopaminergic neurons were detected in the CT-01 hiPSC line (~65%), whereas the lowest numbers were detected in the PD-02 hiPSC line (~13%). The absolute numbers of neurons and dopaminergic neurons that were counted are listed in the Methods section. Statistical power (1−β error probability) >0.85 for all significant conditions. These results suggest that the efficiency of the patterning protocol to generate neurons is indeed not consistent and varies substantially when different hiPSC lines are used.

### Transient overexpression of transcription factors in hiPSCs upon adeno-associated viral vector transduction

**Fig. 2:**
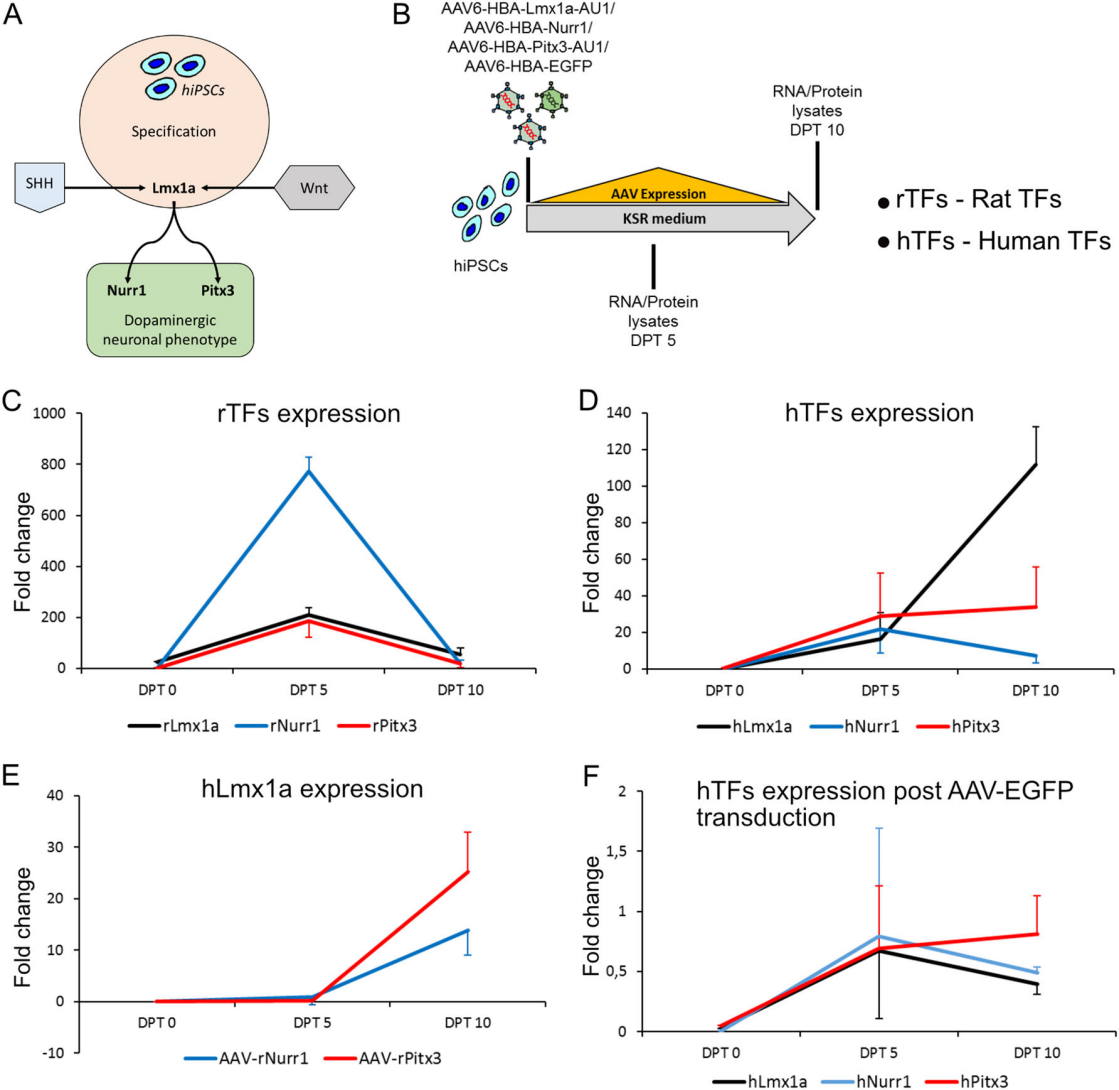
Expression pattern of transcription factors. **a** Schematic representation of involvement of transcription factors Lmx1a, Nurr1 and Pitx3 in patterning of hiPSCs to dopaminergic neurons. **b** Experimental design for the use of viral vector to pattern hiPSCs to generate dopaminergic neurons. **c**–**f** Quantitative analysis of mRNA expression of infected rat transcription factors (rTFs) (**c**), endogenous human transcription factors (hTFs) (**d**), expression of endogenous hLmx1a in hiPSCs after transducing hiPSCs with AAV-HBA-rNurr1 and AAV-HBA-rPitx3-AU1 (**e**) and expression of endogenous human transcription factors (hTFs) after transducing hiPSCs with AAV-HBA-EGFP (**f**). Data represented as fold change ± SD as compared to their respective untreated control at the respective time point from three independent differentiations for each transcription factor.

Many reports have highlighted the importance of controlling certain signaling pathways to successfully pattern hiPSCs to dopaminergic neurons^13,20^. SHH and Wnt signaling pathways indirectly upregulate Lmx1a, which in turn can upregulate dopaminergic neuron-specific transcription factors Nurr1 and Pitx3 (Fig. 2a). We therefore generated viral vectors expressing these transcription factors (Suppl. Fig. 1A). Upon preliminary testing of lentiviral (pLV) versus adeno-associated viral vectors, we found that the latter expressed EGFP much more efficiently in undifferentiated hiPSCs as compared to pLV vectors (Suppl. Fig. 2). Transduction efficiency of lentiviral vector overexpressing EGFP under control of the ubiquitously active Ubiquitin-C (UbC) promoter was observed to be ~20% of hiPSCs at a high viral titer of 10^6^ transducing units (Suppl. Fig. 2A, B). The transduction efficiency of AAV-EGFP under control of the HBA promoter was observed to be around 70–80% at a viral titer of 10^4^ transducing units (Suppl. Fig. 2C, D). In contrast, AAV-EGFP under the control of neuron-specific hSyn1 promoter did not express EGFP in hiPSCs (data not shown), indicating the importance of using an appropriate promoter to drive protein expression in these cells. We first investigated the expression of transcription factors using quantitative real-time polymerase chain reaction (qRT-PCR) (Fig. 2c–f). We used the expression of rTFs, which are almost completely conserved in their amino acids sequences as compared to their human counterparts, but still enabled us to distinguish their mRNAs from endogenous human mRNAs due to variances in their nucleotide sequences. We assessed the expression levels of transcription factors Lmx1a, Nurr1, Pitx3 and EGFP in their respectively transduced cells of the CT-01 hiPSC line at Days in vitro (DIV) 0, DPT 5 and DPT 10 using the schematics shown (Fig. 2b; Suppl. Fig. 3A). In all these post transduction experiments, we observed that mRNA expression levels of the rTFs increased significantly at DPT 5, and were reduced again by DPT 10 (Fig. 2c). This expression pattern was also observed in terms of EGFP expression (Suppl. Fig. 3B). To investigate this issue further, we assessed expression levels of the endogenous human transcription factors (hTFs), after transducing CT-01 hiPSCs with AAVs. We observed that the mRNA expression level of hLmx1a gradually increases when the cells are transduced with AAV-HBA-rLmx1a-AU1 (Fig. 2d). However, the mRNA levels of hNurr1 or hPitx3 did not gradually increase after transducing the cells with AAV-HBA-rNurr1 or AAV-HBA-rPitx3-AU1, respectively (Fig. 2d). After cross-examining the transduced cells at DPT 5 and DPT 10, we observed that the mRNA levels of endogenous hLmx1a gradually increased even when AAV-HBA-rNurr1 or AAV-HBA-rPitx3-AU1 was expressed (Fig. 2e). This was not the case when cells were transduced with a control AAV-HBA-EGFP (Fig. 2f). When cells were transduced with AAV-HBA-rLmx1a-AU1 or AAV-HBA-rPitx3-AU1, we observed the mRNA levels of endogenous hNurr1 to change by an insignificant 0.5–1 fold as compared to the untreated control (Suppl. Fig. 3C). Similarly, after transducing cells with AAV-HBA-rLmx1a-AU1 or AAV-HBA-rNurr1, we observed the mRNA levels of endogenous hPitx3 to change by an insignificant 1–2 fold as compared to the untreated control (Suppl. Fig. 3D). Interestingly, during pharmacological compounds patterning, we observed the endogenous hLmx1a mRNA expression levels to be higher in CT-01 hiPSCs as compared to PD-02 hiPSCs (Suppl. Fig. 3E). We were not able to detect overexpression of rTFs at the same time points by Western blot, although the antibodies that were exploited readily detected their target proteins in lysates of primary neurons that were transduced by the respective vectors (Suppl. Fig. 3F). However, we observed the expression level of EGFP protein to gradually decrease from DPT 5 to DPT 10 (Suppl. Fig. 3F). These data together indicate that adeno-associated viral vectors are suited to transiently overexpress transcription factors depending on the promoter used.

### Adeno-associated viral vector-mediated expression of transcription factors generates consistent numbers of dopaminergic neurons

**Fig. 3:**
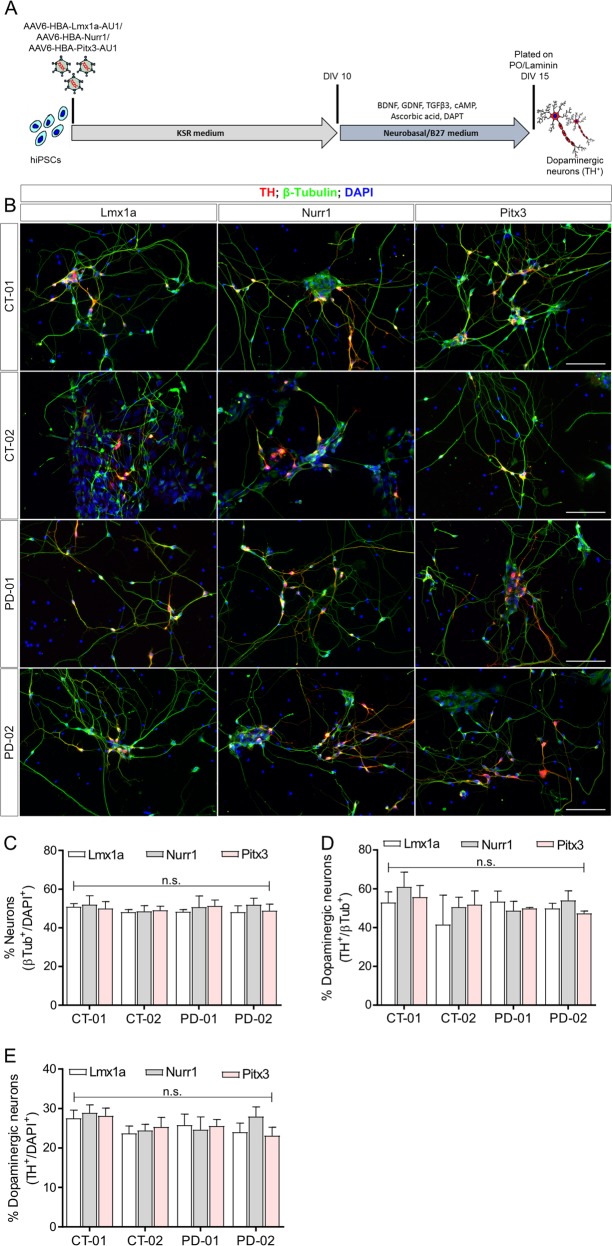
Quantification of percentage of neurons and dopaminergic neurons obtained from four different hiPSC lines using viral vector-mediated patterning. **a** Schematic representation of the use of adeno-associated viral vectors encoding rLmx1a, rNurr1 and rPitx3. **b** Representative fluorescence images of immunoreactivity for the dopaminergic neuronal marker TH (red) and the pan-neuronal marker β-Tubulin (green) at DIV 20. Nuclei are counterstained with DAPI (blue). Scale bars: 100 µm. **c** Quantitative analysis of total percentage of neurons obtained after viral vector-mediated patterning. Data represent the average percentage of total neurons of all cells in culture (β-Tubulin^+^/DAPI^+^). **d** Quantitative analysis of total percentage of dopaminergic neurons obtained after viral vector-mediated patterning. Data represent the average percentage of total dopaminergic neurons out of total number of neurons in culture (TH^+^/β-Tubulin^+^). **e** Quantitative analysis of total percentage of dopaminergic neurons obtained after viral vector-mediated patterning. Data represent the average percentage of total dopaminergic neurons out of total number of cells in culture (TH^+^/DAPI^+^). In all graphs, bars represent the average percentage ± SD from three independent differentiations and independent transductions for each of the four hiPSC lines. Non-significant (n.s.) via two-way ANOVA followed by Bonferroni’s post-hoc test.

To investigate whether expression of any of these transcription factors can efficiently generate dopaminergic neurons, we transduced the four different hiPSC lines with AAVs encoding either rLmx1a, rNurr1 or rPitx3 under the control of HBA promoter (Fig. 3a). The cells were plated at DIV 15 and stained for the neuronal marker β-Tubulin and the dopaminergic marker TH at DIV 20 (Fig. 3b). Upon quantification from at least three independent differentiations and independent transductions for each hiPSC line, we observed that transduction with rLmx1a, rNurr1 or rPitx3 gave rise to similar number of total β-Tubulin^+^ neurons (~50% of all cells in culture; Fig. 3c) and total TH^+^ dopaminergic neurons (~27–30% of all cells; Fig. 3d, e). The absolute numbers of neurons and dopaminergic neurons that were counted are listed in the Methods section. We did not observe any differentiated neurons when hiPSCs were transduced with a control AAV-HBA-EGFP viral vector alone at the same time points. Interestingly, the addition of two or more transcription factors in combination with each other did not result in higher amounts of differentiated neurons or dopaminergic neurons (Suppl. Fig. 4A, C, E, F). In addition, overexpressing these transcription factors along with the pharmacological compounds did not enhance the number of differentiated neurons or dopaminergic neurons in CT-01 hiPSC line as compared to pharmacological compounds patterning alone (Suppl. Fig. 4B, D, G–J).

### Differentiated dopaminergic neurons are functional and release dopamine

**Fig. 4:**
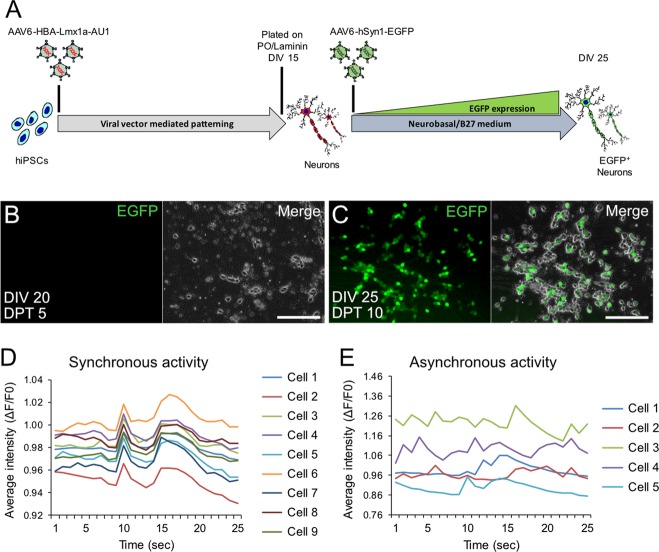
Maturation of hiPSC-derived neurons obtained after viral vector-mediated patterning. **a** Experimental design to monitor expression of EGFP in neurons obtained after rLmx1a viral vector-mediated patterning. **b**, **c** Representative live cell images showing EGFP expression (**c**) or lack of (**b**) in hiPSC-derived neurons at DIV 25 (**c**) vs DIV 20 (**b**). Scale bar: 100 µm. **d**, **e** Quantitative analysis of endogenous spontaneous activity; synchronous (**d**) or asynchronous (**e**) activity observed after transducing CT-01 hiPSC-derived neurons with AAV-hSyn1-GCaMP3.5-EGFP. Data represented as change in average intensity (Δ*F*/*F*0 of GCaMP3.5-EGFP fluorescence) recorded over 25 s per visual field.

**Fig. 5:**
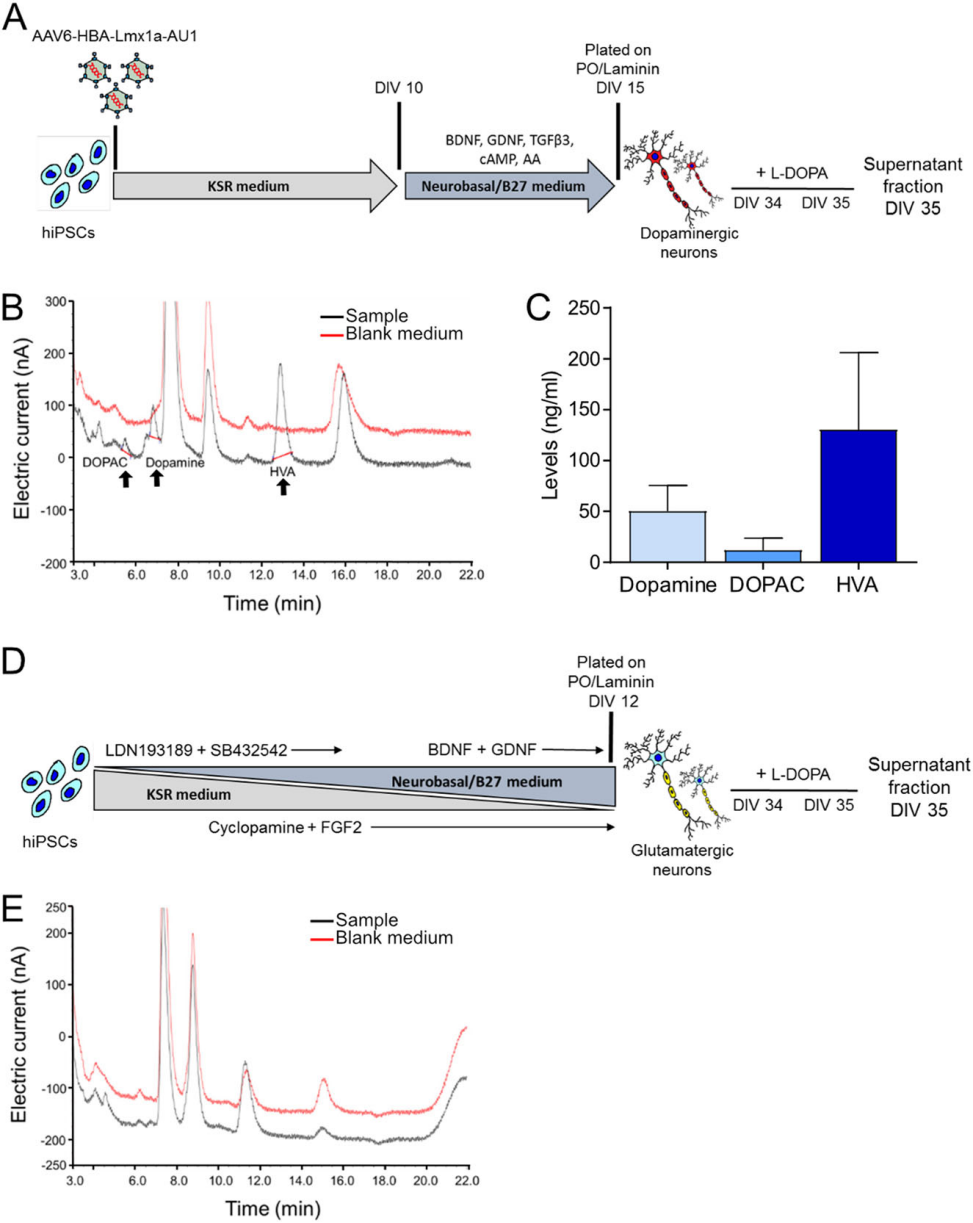
hiPSC-derived neurons release dopamine and its metabolites after treatment with L-DOPA. **a** Experimental design to detect the release of dopamine in CT-01 hiPSC-derived neurons at DIV 35. Neurons were treated with L-DOPA at DIV 34 and DIV 35 with the supernatant collected 4 h later (i.e. at DIV 35). **b**, **c** Representative HPLC chromatogram (**b**) and quantitative analysis (**c**) of released dopamine, DOPAC and HVA in hiPSC-derived dopaminergic neurons patterned using transcription factor rLmx1a. The black and red lines in the chromatogram (**b**) represent supernatant of the sample and matched blank medium (without cells), respectively. **d** Experimental design to detect released dopamine in CT-01 hiPSC-derived glutamatergic neurons at DIV 35. Patterning was performed using SMAD inhibitors LDN/SB along with cyclopamine and FGF2. Neuronal cells were plated on DIV 12 in the presence of growth factors like BDNF and GDNF. Similarly, these neurons were treated with L-DOPA at DIV 34 and DIV 35, with the supernatant collected 4 h later (i.e. at DIV 35). **e** Representative HPLC chromatogram with no detectable levels of dopamine, DOPAC and HVA observed in the supernatant of hiPSC-derived glutamatergic neurons at DIV 35. The black and red lines in the chromatogram (**e**) represent supernatant of the sample and matched blank medium (without cells), respectively. The other unspecific peaks present in the chromatogram (**b**, **e**) arise due to the neurobasal medium composition.

After detecting the neuronal marker β-Tubulin at DIV 20 in CT-01 hiPSC-derived differentiated neurons, we further investigated whether the neurons are mature at this time point. First, we determined when these differentiated neurons would express EGFP under the control of a promoter specific for terminally differentiated neurons, hSyn1. Therefore, after plating cells at DIV 15, we transduced them with AAV-hSyn1-EGFP and monitored EGFP expression over time (Fig. 4a). We noticed that these cells expressed EGFP at DIV 25 (i.e. DPT 10), but not at DIV 20 (i.e. DPT 5), suggesting that they might require a few days in culture to develop neuronal maturity (Fig. 4b, c). Secondly, to determine whether these differentiated neurons are functional, we expressed the GCaMP3.5 calcium sensor and recorded calcium transients as a surrogate for endogenous, non-stimulated electrical activity at DIV 25 (i.e. DPT 10). We observe cells that possessed synchronous (Fig. 4d) as well as asynchronous activity (Fig. 4e). Next, we checked the cell culture supernatant for levels of released dopamine as well as its metabolites DOPAC and HVA, with or without the prior addition of the dopamine precursor L-DOPA, at DIV 35 (Fig. 5a). By HPLC, we observed released dopamine, DOPAC and HVA from the supernatant samples after L-DOPA treatment (Fig. 5b, c). We did not observe any endogenous dopamine release (i.e. without the addition of L-DOPA), which might be below the detection level of our HPLC system. We also did not detect any endogenous dopamine released by neurons obtained after patterning by pharmacological compounds, thereby indicating that these differentiated neurons require longer in culture to release dopamine without stimulation or the released dopamine might be below the detection level of our HPLC system. As an experimental control, we patterned the CT-01 hiPSC line to obtain glutamatergic neurons using the protocol published^21^ (Suppl. Fig. 5A). Upon immunostaining (Suppl. Fig. 5B) and quantification, we obtained ~75% of β-Tubulin^+^ neurons and 65% of CaMKIIβ^+^ glutamatergic neurons out of the total cells in culture (Suppl. Fig. 5C). These differentiated glutamatergic neurons were treated with L-DOPA and the supernatant was analyzed at DIV 35 (Fig. 5d). As expected, we did not observe any released dopamine, DOPAC or HVA from these cultures (Fig. 5e).

### α–synuclein overexpression is toxic to hiPSC-derived dopaminergic neurons

**Fig. 6:**
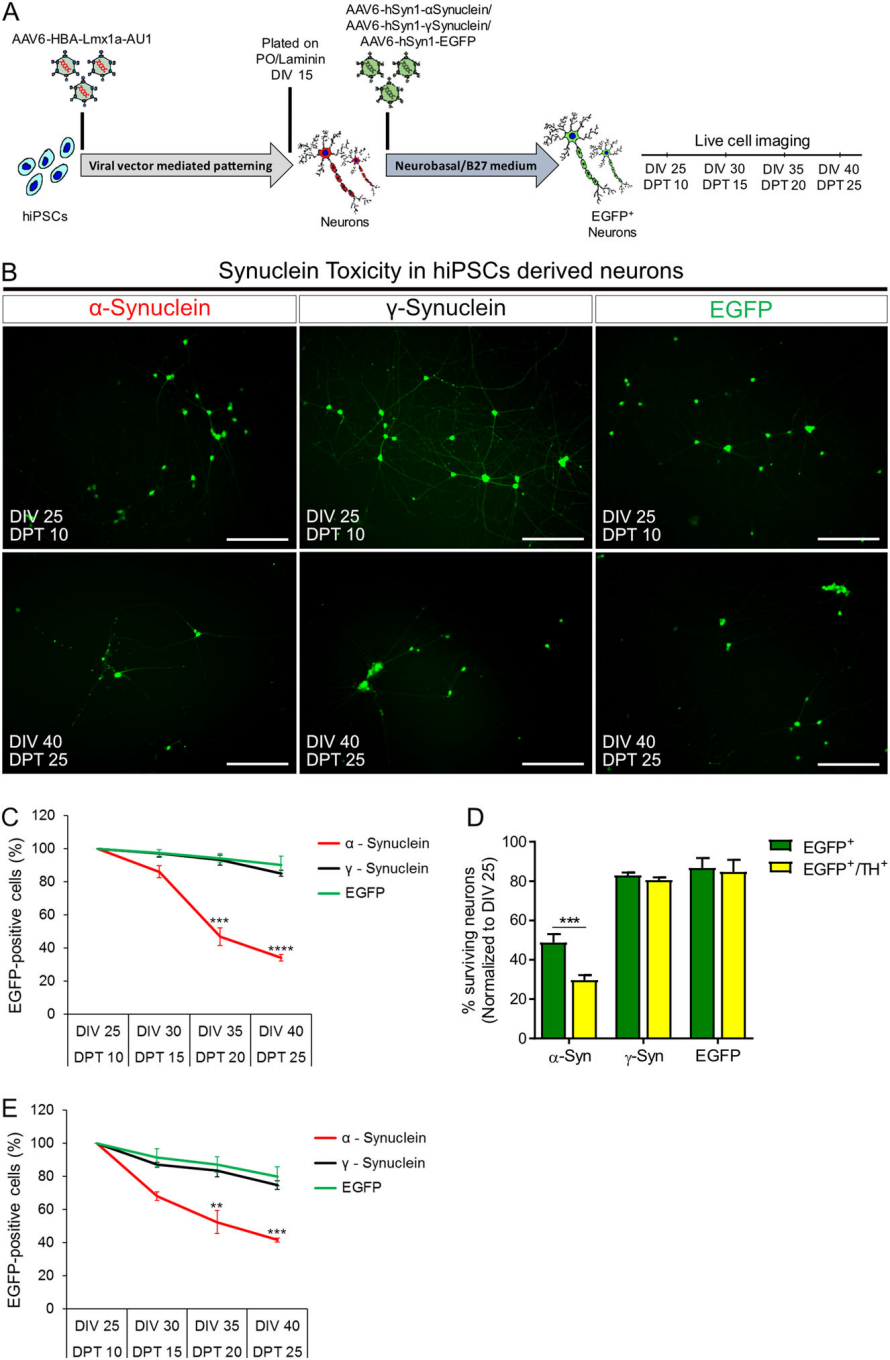
Synuclein toxicity in hiPSC-derived neurons. **a** Experimental design for determining the toxicity induced by synucleins in hiPSC-derived neurons. Confluent CT-01 hiPSCs were transduced with AAV-HBA-rLmx1a-AU1 and kept in culture until DIV 15, when they were plated on coverslips. These neuronal-like cells were transduced with neuron-specific AAV-hSyn1-α-synuclein-EGFP, AAV-hSyn1-γ-synuclein-EGFP or AAV-hSyn1-EGFP viral vector as a control 4 h after plating (i.e. at DIV 15). Live cell imaging was performed every fifth day starting from DIV 25 (i.e. DPT 10) until DIV 40 (i.e. DPT 25). **b** Representative live cell images of hiPSC-derived neurons infected with either α-synuclein, γ-synuclein or EGFP at DIV 25 (DPT 10) and DIV 40 (DPT 25). Scale bars: 100 µm. **c** Quantitative analysis of EGFP^+^ neurons surviving over time when infected with either α-synuclein (red), γ-synuclein (black) or EGFP (green). Data represented as percentage of surviving EGFP^+^ neurons over time normalized to the surviving cells at the first time point, i.e. DIV 25 (DPT 10). Lines represent the average percentage ± SD of EGFP^+^ neurons from three independent experiments and three independent transductions at each time point. **d** Quantitative analysis of percent surviving dopaminergic or non-dopaminergic neurons after transducing CT-01 hiPSC-derived neurons with either α-synuclein, γ-synuclein or EGFP at DIV 40 (DPT 25), normalized to the percent surviving neurons at DIV 25 (DPT 10). Data represent the average percentage ± SD of EGFP^+^ or EGFP^+^/TH^+^ neurons from three independent experiments at DIV 40 (DPT 25). **e** Quantitative analysis of hiPSC-derived glutamatergic EGFP^+^ neurons surviving over time when infected with either α-synuclein (red), γ-synuclein (black) or EGFP (green). Data represented as percentage of surviving EGFP^+^ neurons over time normalized to the surviving cells at the first time point, i.e. DIV 25 (DPT 10). Lines represent the average percentage ± SD of EGFP^+^ neurons from three independent differentiations and three independent transductions at each time point. **p < 0.01; ***p < 0.001; ****p < 0.0001; unpaired *t*-test when compared to EGFP infected neurons at the respective time point.

To determine whether the transcription factor-generated dopaminergic neurons could be used for functional studies, we examined the effect of α-synuclein overexpression on their survival. After plating CT-01 hiPSC-derived differentiated cells, we transduced them with either AAV-hSyn1-α-synuclein-EGFP, AAV-hSyn1-γ-synuclein-EGFP or AAV-hSyn1-EGFP (Suppl. Fig. 1B) and performed live cell counts at an interval of 5 days from DIV 25 (i.e. DPT 10) to DIV 40 (i.e. DPT 25) (Fig. 6a). We first determined that our viral vectors overexpress the respective synucleins by Western blot (Suppl. Fig. 6B) and by immunocytochemistry (Suppl. Fig. 6C) when analyzed at DIV 25 (i.e. DPT 10). Higher magnification images confirmed that higher synuclein expression co-localized with EGFP and was present only in α-synuclein transduced neurons (Suppl. Fig. 6C′-C″; D′-D″). After transducing the neurons with either AAV-hSyn1-α-synuclein-EGFP or AAV-hSyn1-γ-synuclein-EGFP, we quantified EGFP-positive cells at different time points (Fig. 6b). We observed that α-synuclein overexpression was neurotoxic to these neurons as compared to γ-synuclein or EGFP overexpression (Fig. 6c). We observed similar toxicity in differentiated neurons from four different hiPSC lines, when α-synuclein was overexpressed, as compared to their respective γ-synuclein or EGFP overexpressing controls (data not shown). We further examined the specific toxicity to dopaminergic neurons in these cultures by staining them with dopaminergic neuronal marker TH. We observed that ~29% of dopaminergic (TH^+^) neurons overexpressing α-synuclein survived at DIV 40, whereas 60% of non-dopaminergic (TH^−^) neurons survived in the same time after α-synuclein overexpression when normalized to the number of neurons at the first time point, i.e. DIV 25 (Fig. 6d). This suggested that TH^+^ dopaminergic neurons in these cultures were specifically vulnerable to neurotoxicity induced by α-synuclein overexpression. To consolidate this finding, we overexpressed α-synuclein in CT-01 hiPSC-derived glutamatergic neurons and found that the neurotoxicity of α-synuclein was significantly less severe as compared to hiPSC-derived dopaminergic neurons (Fig. 6e; difference in toxicity was to the statistical power (1 − *β* error probability) of 0.9). These data together suggest that the transcription factor patterned dopaminergic neurons are more vulnerable to α-synuclein overabundance as compared to differentiated glutamatergic neurons and thus can be used for studies to better understand the specific neurodegenerative mechanisms or neuroprotective strategies.

## Discussion

Human iPSCs have an immense potential to differentiate into a variety of cells types, including dopaminergic neurons. Over the past decade, numerous protocols have demonstrated that either embryonic stem cells (ESCs)^9^ or hiPSCs can be patterned to generate dopaminergic neurons^8,10–12^. Kriks and coworkers, so far, have reported the most efficient differentiation protocol of hiPSCs into dopaminergic neurons. The authors showed that using pharmacological compounds, hiPSCs are patterned to obtain midbrain floor plate cells, which further mature to give rise to TH^+^ dopaminergic neurons. When we performed dopaminergic neuronal patterning using this protocol in four different hiPSC lines, we observed a pronounced variation in the number of differentiated neurons and dopaminergic neurons generated. This variance is a limiting factor for correct interpretation of comparative studies among various hiPSC lines. It is mandatory that similar numbers of dopaminergic neurons are obtained from different hiPSC lines, if the effects of PD disease-related genes on various factors such as degeneration or electrical activity are to be analyzed. To overcome this issue, we decided to express TFs, which are known to be essential for generation of dopaminergic neurons. Although the number of neurons and dopaminergic neurons generated after viral vector-mediated patterning is lower as compared to those generated with pharmacological compounds in some hiPSC lines, we observe very consistent numbers of differentiated dopaminergic neurons in four different hiPSC lines. On contrary to previous publication^22^, we report that overexpression of a single transcription factor i.e. either Lmx1a/Nurr1/Pitx3 is capable of generating ~50% total neurons in culture and 28% of dopaminergic neurons (of total cells).

It has been previously demonstrated that Wnt1 directly regulates Lmx1a, which further regulates the expression of Nurr1 and Pitx3 during early midbrain dopaminergic neuronal differentiation. In contrast, Wnt1 is also reported to be a major target of Lmx1a, thereby indicating the existence of an autoregulatory loop involving Lmx1a and Wnt1^23^. In addition, we here demonstrate that a feedback loop between Lmx1a and Nurr1-Pitx3 exists in hiPSCs, which regulates the expression of Lmx1a, one of the major driving forces in the differentiation process of dopaminergic neurons. Moreover, Foxa2, known to upregulate SHH signaling^20^, also upregulates Lmx1a during the specification of dopaminergic neurons^24^. However, whether there exists an autoregulatory loop between Lmx1a and Foxa2 remains unknown. However, the presence of such a loop might provide an explanation as to why Lmx1a alone is able to pattern the fate of hiPSCs to dopaminergic neuronal phenotype. Interestingly, overexpressing rat Lmx1a/Nurr1/Pitx3, but not EGFP, resulted in an upregulation of human Lmx1a mRNA levels. To our knowledge, this is the first time that a species-related increase of transcription factors expression has been reported in hiPSCs.

Our approach of overexpressing TFs by AAVs under the control of the HBA promoter is self-limiting, but the molecular basis for silencing of the HBA promoter remains to be elucidated. The number of surviving cells remain constant over time (data not shown), and thus it is unlikely that the vector genome becomes diluted through cell division. Targeted DNA methylation could thus have silenced the HBA promoter^25^.

One of the most important points, which needs to be addressed in the field, is the time required for these differentiated neurons to mature in culture. Even though the differentiated cells express the neuronal marker β-Tubulin at day 20, they are not necessarily mature. We, therefore, used EGFP expression under the control of hSyn1 promoter as a surrogate marker system. Activity of the hSyn1 promoter is controlled by the RE-1 silencing transcription factor (REST) by binding to a specific site, thereby silencing the promoter^26^. When compared to progenitor cells, the expression of REST is downregulated in mature neurons^27^ and therefore the hSyn1 promoter is only active in mature cells. We report that in our culture system, the differentiated neurons require at least 10 days to mature and express EGFP from a hSyn1 promoter. The time required for maturation was confirmed by recording synchronous and asynchronous electrical activity at day 25. We detected released dopamine and its metabolites DOPAC and HVA in the supernatant at day 35 after the addition of L-DOPA, suggesting these neurons require more time in culture to mature, form neural networks and build dopamine producing machinery, than previously thought.

We also observe neurotoxicity in hiPSC-derived dopaminergic neurons and glutamatergic neurons when α-synuclein is overexpressed. It is interesting that α-synuclein overexpression is specifically more toxic to differentiated dopaminergic neurons as compared to differentiated glutamatergic neurons, suggesting the need to explore the unknown mechanisms, which make dopaminergic neurons more vulnerable to α-synuclein toxicity. Together, these data indicate that the differentiated neurons could be successfully used for further experiments within the culturing time frame. The viral vector-mediated patterning enabled us to perform reasonable neurotoxicity studies in different hiPSC lines.

In conclusion, we report a simple and reliable method of patterning hiPSCs to dopaminergic neurons with a major advantage being the homogeneity of differentiated neurons derived from multiple hiPSC lines. Moreover, we report that hSyn1 promoter-driven expression of a reporter gene could be used as a surrogate maturation marker. We also provide insight in the regulatory role of human Lmx1a and Nurr1-Pitx3, which are the major transcription factors required in the patterning of dopaminergic neurons.

## Experimental procedures

### Generation of hiPSCs

Gingival fibroblasts from a 32-year-old female with no apparent health conditions were used to generate the line by Sendai virus reprogramming. This line, hiPS-G1, has been reported^28^. This line is termed as CT-01 in this manuscript. The other lines used in this manuscript were generated from dermal skin fibroblasts of PD patients or control individuals by Sendai virus reprogramming. Line CT-02 is generated from a 57-year-old female with no clear health conditions. The first idiopathic PD-01 line is generated from a 72-year-old male (age at disease onset—66 years). The second idiopathic PD-02 (with ICD) line is obtained from a 63-year-old male (age at disease onset—54 years).

### Pharmacological compounds driven dopaminergic neuronal patterning

Dopaminergic neural induction was performed using pharmacological compounds as published^13^. Cells were plated on matrigel (BD Biosciences; 354320) coated wells and cultured for 10 days in neural induction medium containing knockout DMEM serum replacement medium (Invitrogen; 10829–018) supplemented with 15% knockout serum replacement (Invitrogen; 10828010), 2 mM l-Glutamine (Invitrogen; 25030–081) and 10 µM β-mercaptoethanol (Invitrogen; 31350–010). SMAD inhibitors LDN193189 (100 nM; Stemgent; 04-0074-10) and SB431542 (10 µM; Tocris; 1614) were added from day 0 to day 7. Upregulation of SHH was induced by adding a combination of SHH C25II (100 ng/ml; R&D systems; 464-SH-200/CF), purmorphamine (2 µM; Stemgent; 04-0009) and FGF8 (100 ng/ml; R&D systems; 423-F8-025/CF) from day 1 to day 6. Inhibition of WNT was induced by adding a GSK3β inhibitor, CHIR99021 (3 µM; Stemgent; 04-0004-10) from day 3 to day 13. On day 11, the medium was changed to differentiation medium containing Neurobasal (Invitrogen; 21103-049) supplemented with 1% B27 (Invitrogen; 10889-038), 2 mM l-Glutamine and growth factors like BDNF (20 ng/ml; R&D systems; 248-BD-025/CF), GDNF (20 ng/ml; R&D systems; 212-GD-010/CF), TGFβ3 (1 ng/ml; R&D systems; 243-B3-002/CF), dibutyryl cAMP (0.5 mM; Sigma; D0260), ascorbic acid (0.2 mM; Sigma; A4403) and DAPT (10 µM; Tocris; 2634) till day 20. On day 20, the cells were dissociated using Versene (Thermo Scientific; 15040-033) and re-plated in wells with or without coverslips, pre-coated with poly-L-ornithine (50 µg/ml; Sigma; P-3655) and laminin (2 µg/ml; Sigma; L2020). The cells were maintained in the differentiation medium until analysis. Upon staining and quantification, we observed the absolute number of total DAPI^+^ cells per square-millimeter for each line: CT-01 (403 ± 76), CT-02 (208 ± 59), PD-01 (235 ± 48) and PD-02 (230 ± 35). The absolute numbers of β-Tubulin-positive neurons per square-millimeter quantified for each line: CT-01 (286 ± 33), CT-02 (117 ± 42), PD-01 (100 ± 19) and PD-02 (102 ± 27). The absolute numbers of TH-positive dopaminergic neurons per square-millimeter quantified for each line: CT-01 (213 ± 43), CT-02 (57 ± 27), PD-01 (44 ± 11) and PD-02 (52 ± 19).

### Construction and propagation of viral vectors

Recombinant adeno-associated viral vectors of serotype 6 (AAV-6) were prepared by transient transfection of vector genome plasmids (Suppl. Fig. 1) with the DP6 helper plasmid in HEK293 cells. Viral particles were purified from cell lysates by iodixanol gradient centrifugation and heparin affinity chromatography. These viral vectors overexpressed the transcription factors under the control of the HBA promoter, as previously reported^29^. After extensive dialysis against PBS particles were frozen in single-use aliquots at −80 °C. Genome titers were determined by qPCR and >98% purity was confirmed by SDS-PAGE. Vector genome (vg) titer versus transducing units (tu) titer was determined with EGFP expressing vectors in primary neurons and was estimated to be 1:30 (tu:vg).

### Viral vector-mediated dopaminergic neuronal patterning

Viral vectors encoding rat Lmx1a, Nurr1 and Pitx3 were added in combination with each other or separately to hiPSCs at a confluent state. Base knockout DMEM medium supplemented with 15% knockout serum replacement, 2 mM l-Glutamine and 10 µM β-mercaptoethanol was added from day 0 to day 10. The medium was kept unchanged for the first 2 days to enable efficient viral infection. From day 11 onward, the medium was 100% replaced every day. At day 11, the medium was changed to differentiation medium containing Neurobasal supplemented with 1% B27, 2 mM l-Glutamine and growth factors like BDNF, GDNF, TGFβ3, dibutyryl cAMP, ascorbic acid and DAPT until day 15. The cells were then dissociated using Versene and replated in wells with or without coverslips, pre-coated with poly-l-ornithine and laminin. The cells were maintained in complete medium until analysis. Upon staining and quantification, we observe the absolute number of total DAPI^+^ cells per square-millimeter for each line: CT-01 (190 ± 48), CT-02 (137 ± 26), PD-01 (185 ± 26) and PD-02 (151 ± 32). The absolute numbers of β-Tubulin positive neurons per square-millimeter quantified for each line: CT-01 (107 ± 36), CT-02 (72 ± 16), PD-01 (93 ± 20) and PD-02 (75 ± 17). The absolute numbers of TH-positive dopaminergic neurons per square-millimeter quantified for each line: CT-01 (60 ± 19), CT-02 (36 ± 9), PD-01 (47 ± 9) and PD-02 (38 ± 10). Glutamatergic neurons (CAMKIIβ^+^; 27.92 ± 4.85% of all cells) and astrocytes (6.54 ± 1.9% of all cells) were detected in these cultures. The differentiated neurons were also positive for other dopaminergic markers such as AADC and DAT (data not shown).

### Pharmacological compounds driven glutamatergic neuronal patterning

The glutamatergic neuronal induction protocol was modified and performed using pharmacological compounds as published^21^. CT-01 hiPSCs were plated on Matrigel coated wells and cultured for 12 days. The neural induction medium consisted of knockout DMEM medium, 15% knockout serum replacement and 2 mM l-Glutamine that was gradually changed to Neurobasal, 1% B27 and 2 mM l-Glutamine. SMAD signaling inhibitors LDN193189 (1 µM) and SB432545 (10 µM) were added to the medium from day 1 to 7 of neural induction. Cyclopamine (400 ng/ml; Calbiochem; 239804) and FGF-2 (10 ng/ml; Peprotech; 100-18B) were added to the medium from day 3 to 12. At Day 12, the cells were dissociated using Versene and re-plated in wells with or without coverslips, pre-coated with poly-l-ornithine and laminin. The cells were maintained in the differentiation/maturation medium until analysis. The differentiation/maturation medium consisted of Neurobasal, 1% B27, 2 mM l-Glutamine and growth factors like 20 ng/ml BDNF, 20 ng/ml GDNF and 10 µM DAPT.

### Antibodies

The following primary antibodies were used for the experiments: mouse monoclonal antibodies against β-Tubulin, clone TUB 2.1 (Sigma; T4026; 1:500), anti-Nurr1 (Abcam; ab41917; 1:1000) and anti-GFP (Roche; 11814460001; 1:10,000); rabbit polyclonal antibodies against TH (Millipore; AB152; 1:250), anti-Myc (Cell Signaling; 2272; 1:1000), anti-Pitx3 (Abcam; ab30734; 1:1000), anti-pan-synuclein (Abcam; ab6176; 1:500), anti-γ-synuclein (Abcam; ab6169; 1:1000) and anti-CaMKIIβ (Abcam; ab34703; 1:1000). For immunocytochemistry, secondary antibodies conjugated with Cy2/Cy3 fluorophores (Dianova; 115-225-072/111-165-006; 1:500) were used. Nuclear counterstaining was done with 4′, 6′-diamidino-2-phenylindole (DAPI; 2 µg/ml; Invitrogen; D3571). For Western blot, anti-mouse HRP (Dianova; 715-035-150; 1:5000) or anti-rabbit HRP (Dianova; 711-035-152; 1:5000) secondary antibodies were used. Protein levels were normalized to Actin clone c4 (Millipore; MAB1501).

### Immunocytochemistry

Immunocytochemistry was performed as previously described with minor modifications^30^. Briefly, 4% paraformaldehyde (PFA) dissolved in PBS was used to fix cells for 20 min at room temperature. Later, cells were washed thrice with PBS for 5 min each. Further, cells were blocked and permeabilized with 10% normal goat serum (GeneTex; GTX73206) in PBS containing 0.25% Triton X-100 for 45 min. Coverslips were incubated with primary antibodies diluted in blocking solution overnight at 4 °C. The cells were further washed thrice with PBS for 5 min each, followed by incubation with Alexa dye-conjugated secondary antibodies for at least 2 h at room temperature. After washing thrice with PBS for 5 min each, coverslips were incubated in PBS containing DAPI for 5 min at room temperature. Three PBS washes later, the coverslips are mounted on slides using Mowiol 4–88 mounting medium (Sigma; 81381). Fluorescence images were collected using an automated Zeiss Axioplan 2 microscope, equipped with a Zeiss AxioCam ERc5s camera and 20× or 40× Plan—NEOFLUAR or 63× Plan—APOCHROMAT objectives and Zeiss AxioVision V4.8.2.0 software. The numbers of total cells (DAPI^+^), neurons (β-Tubulin^+^), dopaminergic neurons (TH^+^) and glutamatergic neurons (CAMKIIβ^+^) were counted manually and percentages of total neurons (β-Tubulin^+^/DAPI^+^), total dopaminergic neurons (TH^+^/DAPI^+^) and total glutamatergic neurons (CAMKIIβ^+^/DAPI^+^) were plotted using ImageJ v1.51 software. The same image acquisition parameters and analysis methods were used for quantification of the number of neurons, dopaminergic neurons and glutamatergic neurons from all the patterning protocols.

### Dopamine detection by HPLC with electrochemical detection

Medium supernatant and intracellular fraction from CT-01 hiPSCs-derived dopaminergic or glutamatergic neurons were collected at DIV 35. Buffer containing 2 M perchloroacetic acid (PCA) and 1% sodium metabisulfite was used to stabilize the released dopamine from the supernatant fraction. This fraction was filtered through a Minisart® SRP4 filter (0.4 µm; Sartorius; 17820) and 20 µl was then injected into the HPLC system. The HPLC system is supplemented with a pump and automated injector along with Acclaim 120, C8 analytical column (5 µm; 2.1 × 250 mm; Thermo Scientific; 059137) and a two-channel Coulochem II ESA electrochemical detector (ECD). For dopamine, DOPAC and HVA quantification, the mobile phase used contained 85 mM sodium acetate, 38.3 mM citric acid, 0.16 mM EDTA, 0.49 mM sodium octane sulfonic acid and 10% methanol, at pH 4.50. The flow rate was maintained at 0.4 ml/min and the signal was detected at 50 mV and 300 mV. The obtained values were quantified as per the corresponding standards containing 0.15 µM, 0.30 µM and 1.5 µM of dopamine, DOPAC and HVA, respectively. The values were analyzed using Chromeleon v6.80 software.

### Endogenous calcium imaging

CT-01 hiPSC-derived neurons were transduced with the EGFP-based genetically encoded calcium indicator (GECI) GCaMP3.5 as previously reported^31^ and Ca^2+^ live imaging was performed at DIV 25 using a Zeiss AxioVert observer Z1 microscope equipped with an AxioCam 503m_S815 camera and imaged using Zen pro v2.3.64 software. The calcium influx was measured using hand drawn ROIs around whole cells. Around 21 ± 6 and 12 ± 3 neurons per square-millimeter showed synchronous and asynchronous activity, respectively. Data were analyzed using ImageJ v1.51 software and the change in intensity (Δ*F*/*F*0) was plotted using GraphPad prism v6.01 software.

### Synuclein toxicity in hiPSC-derived neurons

CT-01 hiPSC-derived neurons were plated on poly-l-ornithine and laminin-coated wells. Synuclein overexpressing viral vectors (α- and γ-synuclein) along with a control EGFP viral vector (viral titer 10^8^ tu/well) were added to their respective wells 4 h after plating. Viral vectors overexpressing α- or γ-synuclein or EGFP were generated as mentioned above, with the expression under the control of a neuron-specific hSynapsin1 gene promoter (Suppl. Fig. 1B). EGFP expression was visible at 10 DPT. Live cell imaging was performed at DPT 10, 15, 20 and 25 from the same wells. Surviving EGFP-positive cells were quantified manually and plotted using GraphPad prism v6.01 software. For experiments addressing toxicity of synucleins in dopaminergic against non-dopaminergic neurons, the cultures were stained with a dopaminergic neuronal marker (TH) and EGFP. Images were recorded and the number of neurons expressing either EGFP or TH and EGFP alone were manually quantified and plotted using GraphPad prism software.

### Primary cortical neuronal culture

Neuronal cultures were prepared from embryonic day 17.5 rat pups as previously described^32^. In short, the cortical plate is isolated from the embryos and dissociated into single cells using 0.25% Trypsin (Gibco; 15090-046). The cells are maintained at 37 °C for 15 min. The cells are then plated in 24-well plates pre-coated with poly-l-ornithine (50 µg/ml; Sigma; P-3655) and laminin (2 µg/ml; Sigma; L2020) in Neurobasal medium (750 µl/well; Invitrogen; 21103-049) supplemented with 1% penicillin–streptomycin–neomycin (PSN; Gibco; 15640-055), l-Glutamine (0.5 mM; Invitrogen; 25030-081), 0.5% transferrin (AppliChem; A3124-0250) and 2% B27 serum (Invitrogen; 10889-038), until analysis. For Western blot, cells are plated at a density of 250,000 cells per well. Transduction with viral vectors (titer 10^8^ tu/well) were performed at DIV 0 (for Lmx1a and Pitx3) and DIV 3 (for Nurr1) and the cells were lysed at DIV 5 (for Lmx1a and Pitx3) and DIV 10 (for Nurr1) to determine overexpression.

### Quantitative real-time PCR

Undifferentiated CT-01 hiPSCs at different time intervals (DIV 0, DPT 5 and DPT 10) were collected by centrifugation. Cell pellets were stored at −80 °C until further analyzed. RNA was extracted from the pellets using the TRI Reagent (Sigma; T9424) and quantified with NanoDrop One (Thermo Scientific). Total RNA was reverse transcribed using QuantiTect-Reverse Transcription kit (Qiagen; 205311) and was analyzed by qRT-PCR (25 ng RNA equivalent/reaction) using target-specific primers (Suppl. Table 1) and QuantiFast SYBR Green PCR kit (Qiagen; 204143). Fifty qRT-PCR cycles were performed (with denaturing and annealing done at 94 °C and 55 °C, respectively) on QuantStudio 3 (Applied Biosystems) machine equipped with the QuantStudio^TM^ Design & Analysis software. Data were normalized using the expression of the housekeeping genes β-Actin and GAPDH. Ct values were converted into fold-expression values relative to the control (untreated at respective time point) using QuantStudio^TM^ Design & Analysis v1.4 software.

### Western blotting

Cells were lysed by sonication in lysis buffer containing 50 mM TRIS pH 8.0, 0.5% SDS and 1 mM DTT supplemented with protease inhibitors (Roche; 11697498001). Equal amounts of protein (20 µg) were separated on 10–12% bis-tris polyacrylamide gels and transferred on PVDF membranes (0.45 µm; AppliChem; A5243-3030R) and further incubated with corresponding antibodies at 4 °C overnight. The membranes were washed thrice with TBST (with 0.25% Triton X-100) and incubated with the respective secondary antibodies for 2 h at room temperature. Lab-made ECL was used and protein detection was performed with the help of X-ray detection system. Equal loading was verified using MemCode (Thermo Scientific; 24580) as previously described^32^.

### Statistical analysis

All experiments were performed with at least three independent differentiations and/or three independent transductions where each consisted of at least three biological replicates of which each consisted of three technical replicates. Experiments were replicated until the statistical power was observed to be >0.80 (1−β error probability > 0.80) using G*Power v3.1.9.2. Statistical analysis was performed using GraphPad Prism v6.01 software. For datasets with normal distribution, the Student’s *t*-test was used for comparison between two groups. One-way or two-way ANOVA followed by Bonferroni’s post-hoc test was used for comparing more than two groups at a specific time interval. The differences between groups were considered statistically significant when *p* < 0.05.

## Supplementary information

Supplementary Figure 1

Supplementary Figure 2

Supplementary Figure 3

Supplementary Figure 4

Supplementary Figure 5

Supplementary Figure 6

Supplementary Figure Legends

Supplementary Table
