# Supplementary figures and images for: Homogenous generation of dopaminergic neurons from multiple hiPSC lines by transient expression of transcription factors
Source: Cell Death Dis. 2019 Nov 27;10(12):898. doi: 10.1038/s41419-019-2133-9 (PMC6881336; doi:10.1038/s41419-019-2133-9)

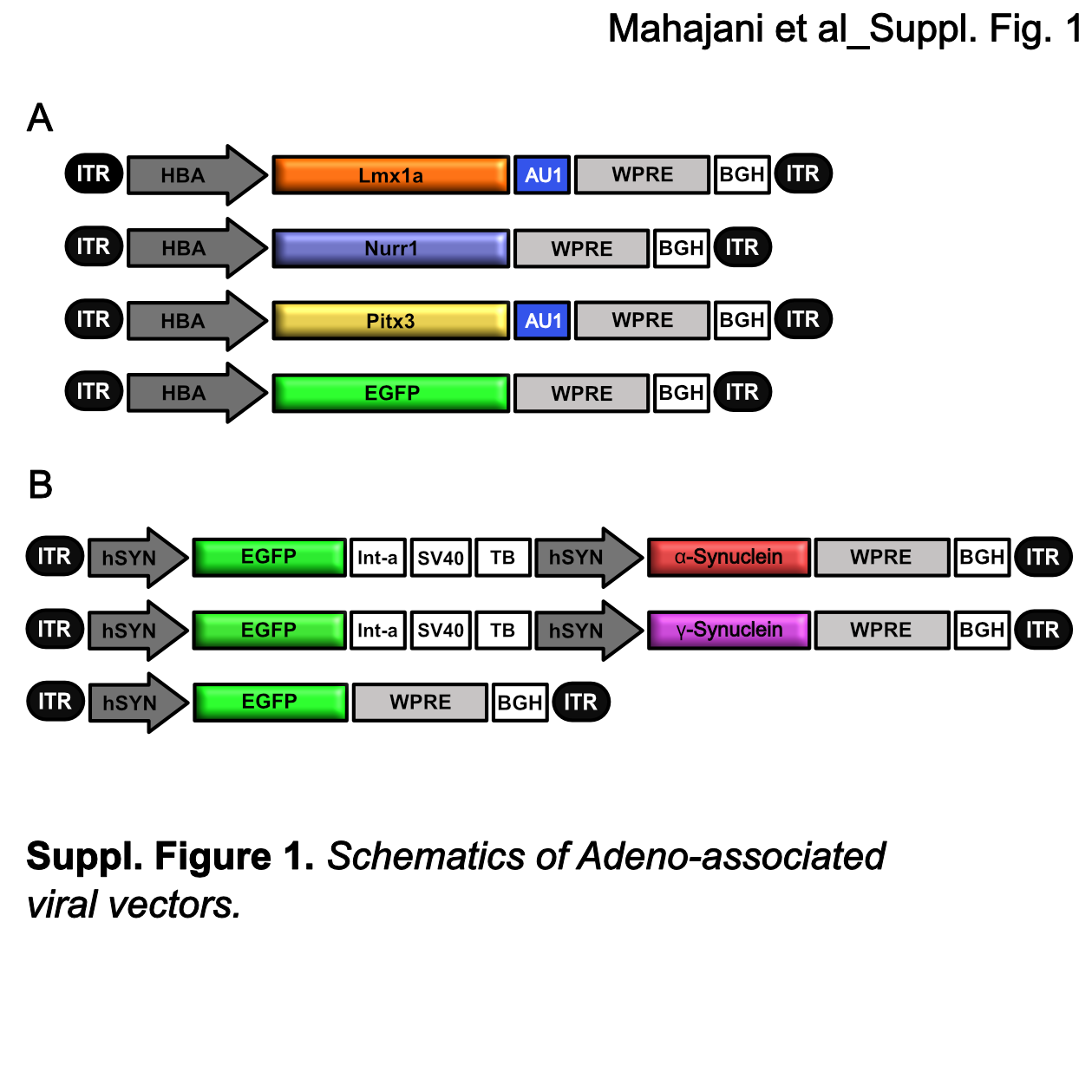

Supplement: Supplementary file 1 — Supplementary Figure 1 [file 41419_2019_2133_MOESM1_ESM.tif]

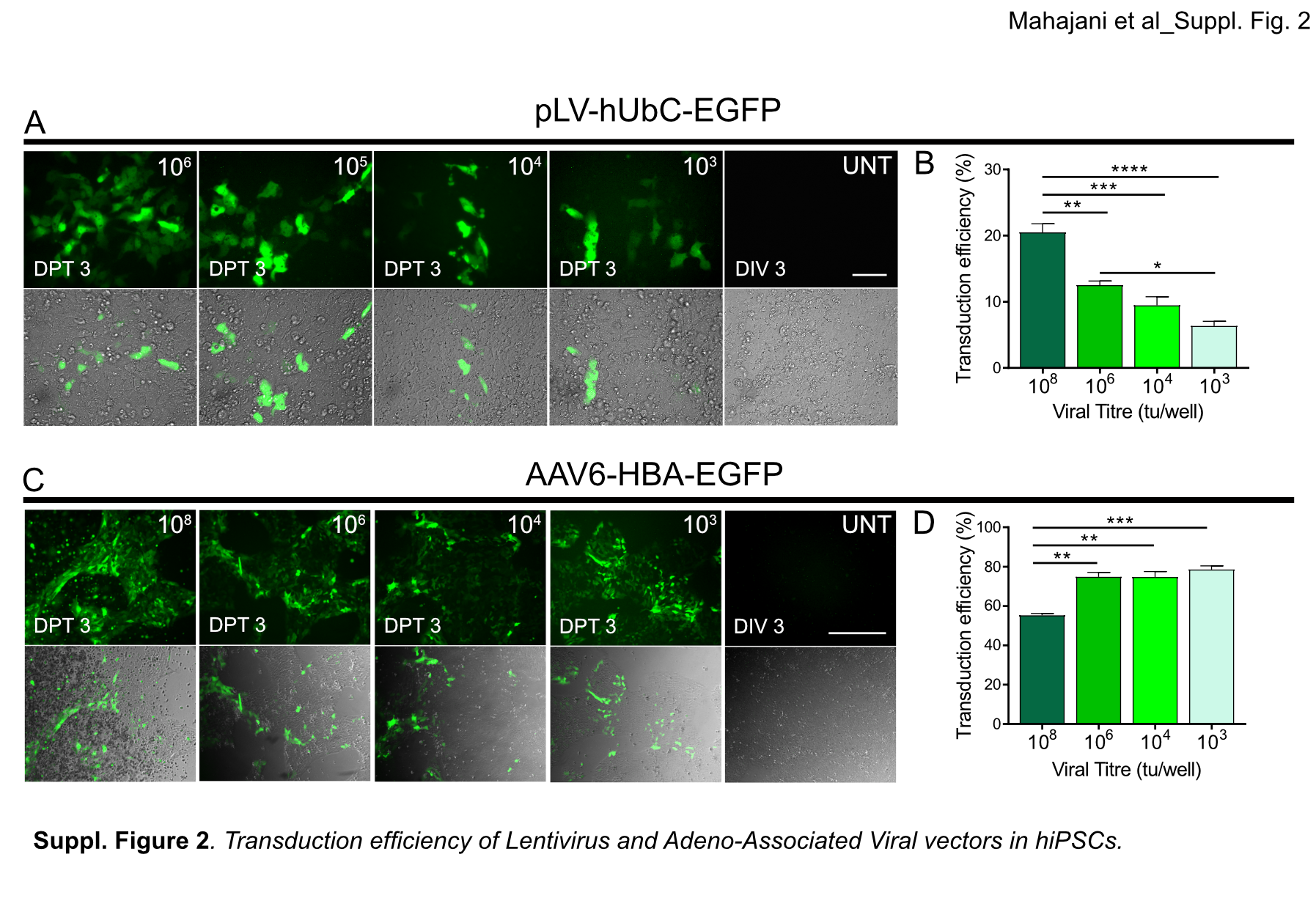

Supplement: Supplementary file 2 — Supplementary Figure 2 [file 41419_2019_2133_MOESM2_ESM.tif]

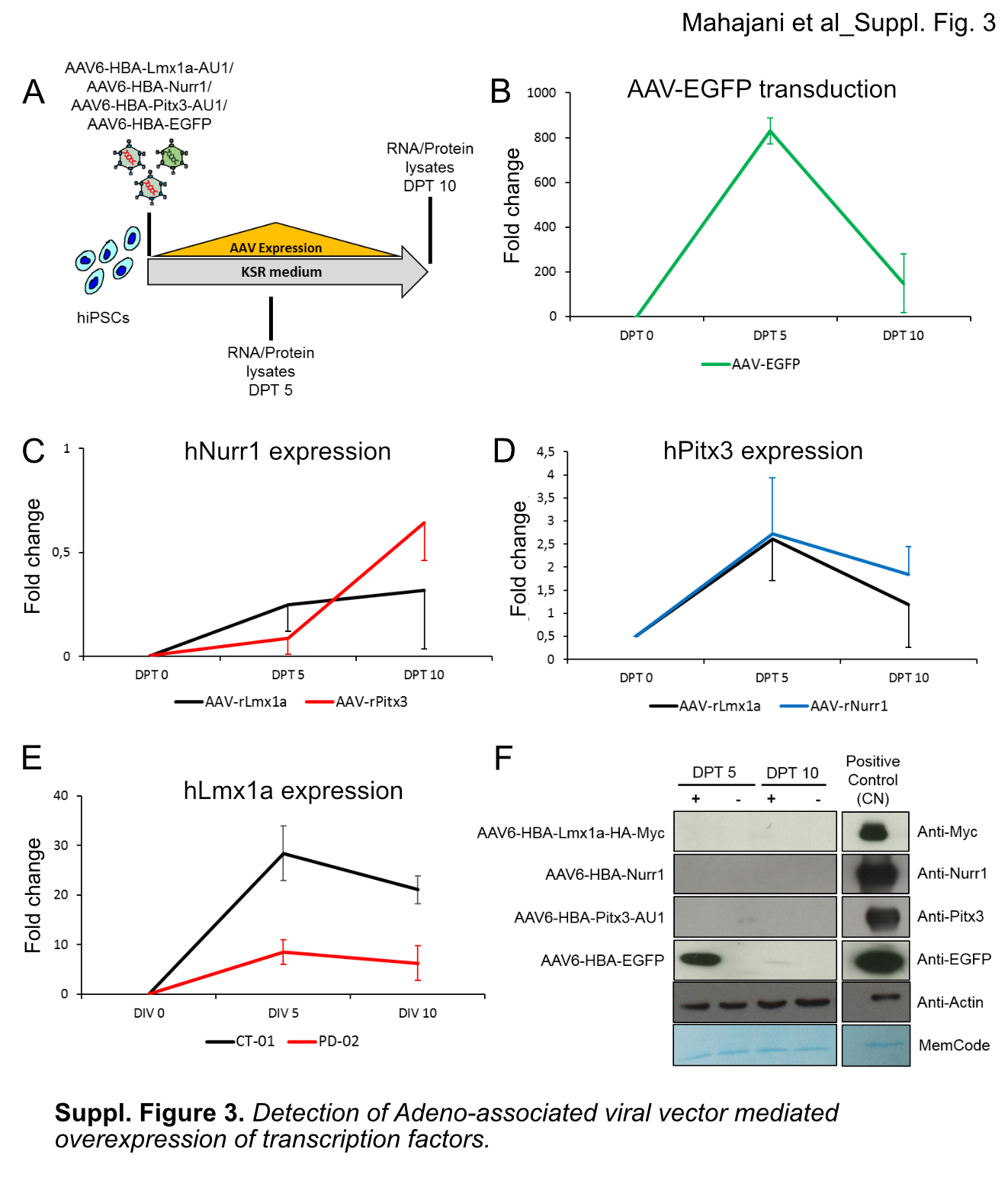

Supplement: Supplementary file 3 — Supplementary Figure 3 [file 41419_2019_2133_MOESM3_ESM.tif]

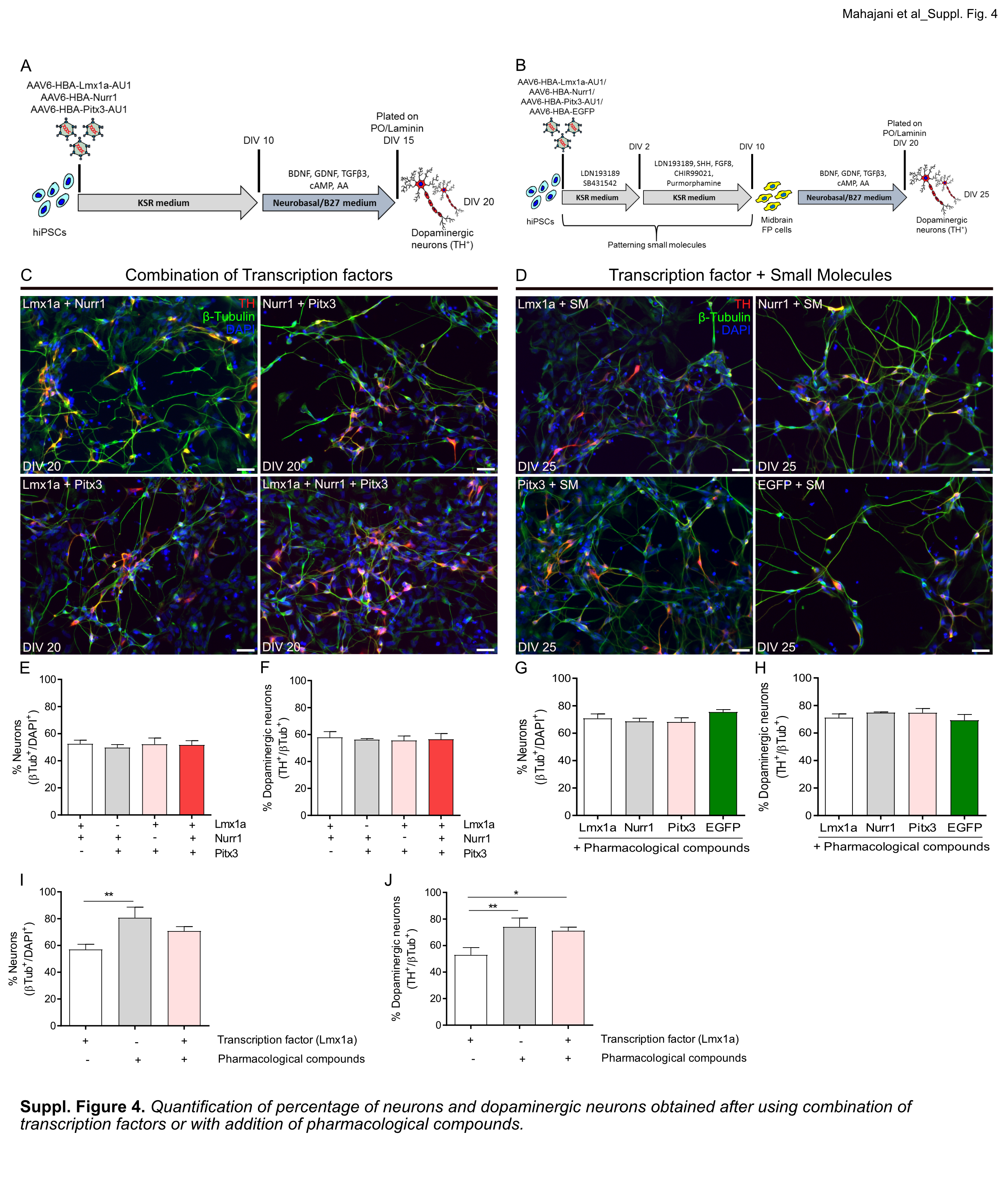

Supplement: Supplementary file 4 — Supplementary Figure 4 [file 41419_2019_2133_MOESM4_ESM.tif]

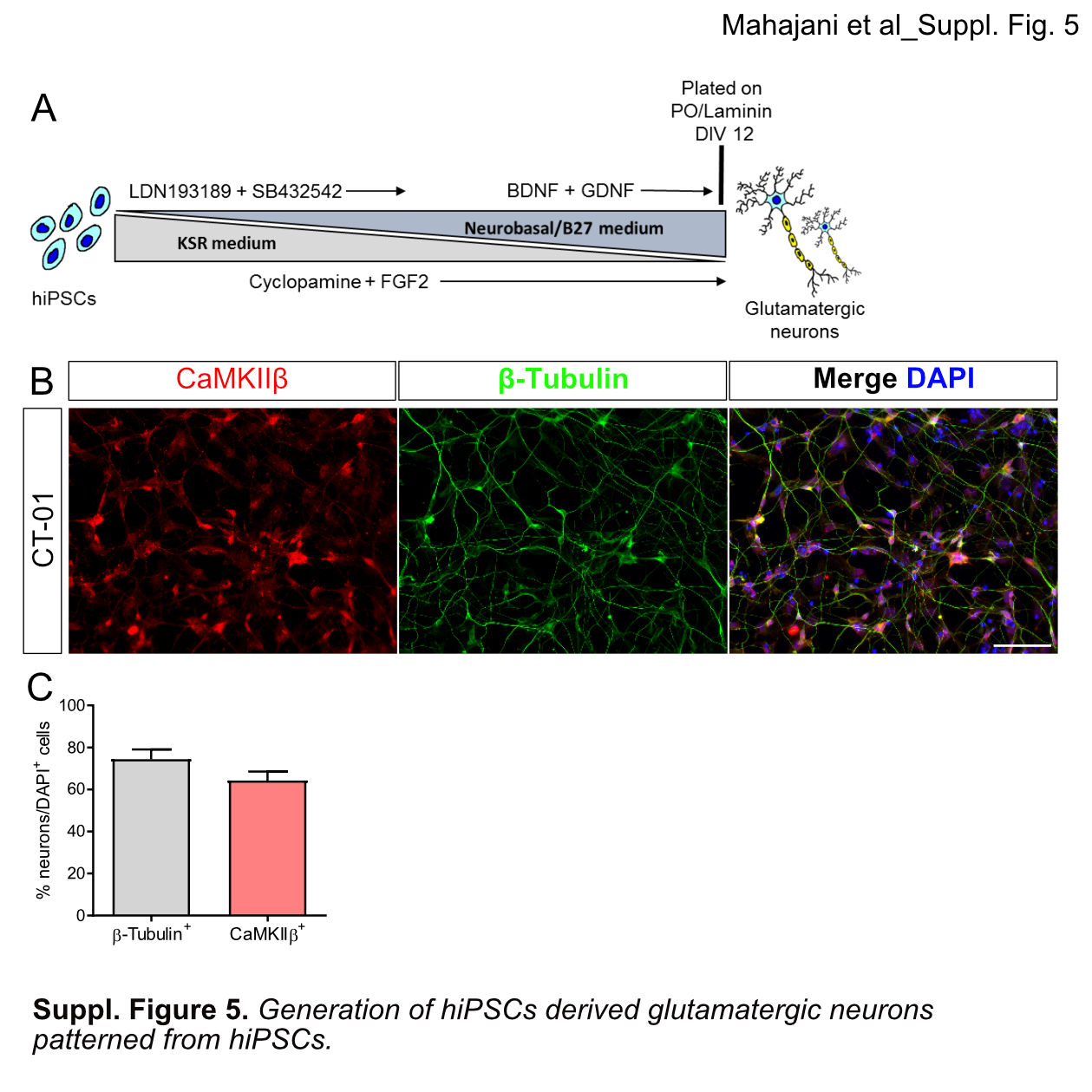

Supplement: Supplementary file 5 — Supplementary Figure 5 [file 41419_2019_2133_MOESM5_ESM.tif]

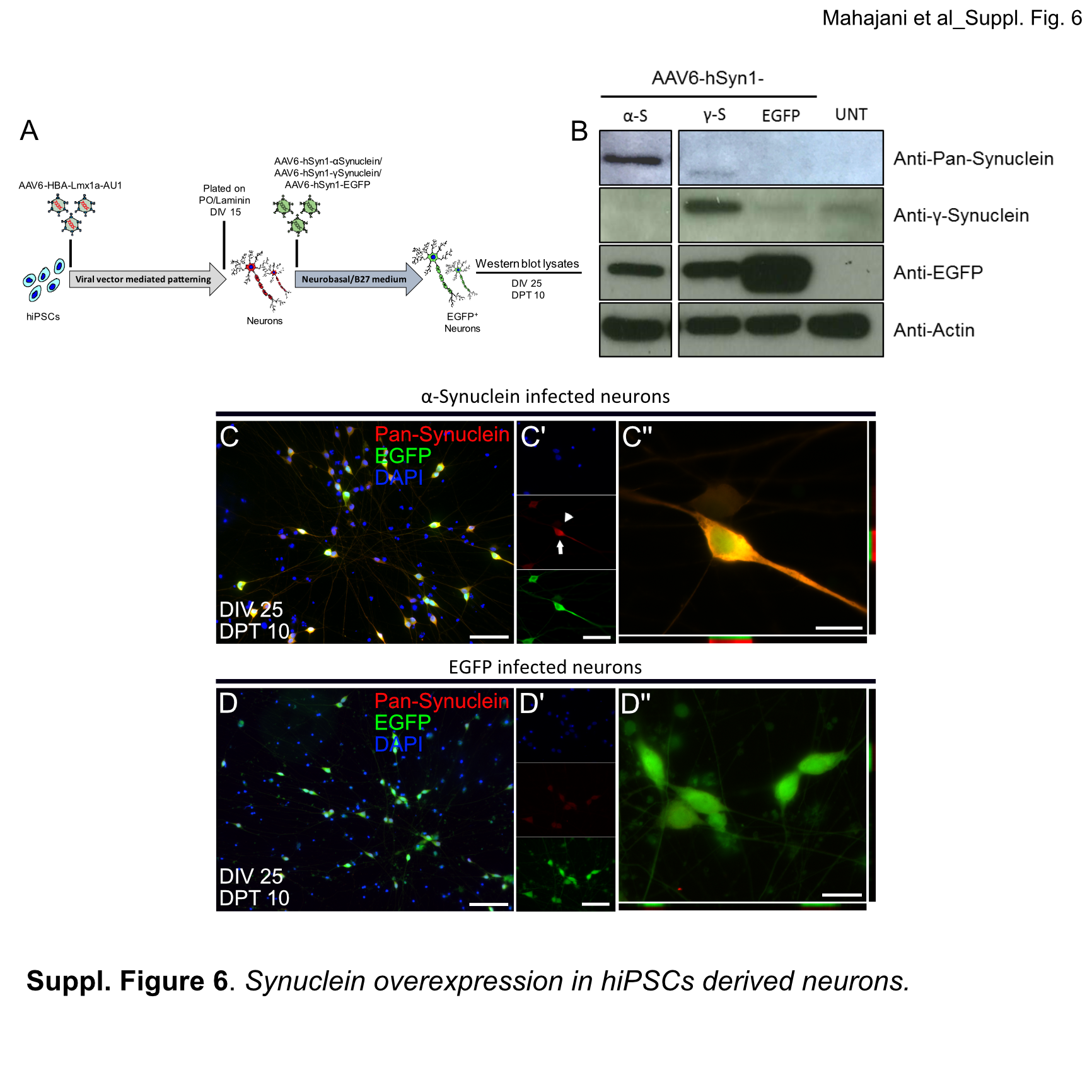

Supplement: Supplementary file 6 — Supplementary Figure 6 [file 41419_2019_2133_MOESM6_ESM.tif]
