# Supplementary material for: Homogenous generation of dopaminergic neurons from multiple hiPSC lines by transient expression of transcription factors
Source: Cell Death Dis. 2019 Nov 27;10(12):898. doi: 10.1038/s41419-019-2133-9 (PMC6881336; doi:10.1038/s41419-019-2133-9)
Supplement: Supplementary file 7 — Supplementary Figure Legends [file 41419_2019_2133_MOESM7_ESM.docx]

**Homogenous generation of dopaminergic neurons from multiple hiPSCs lines by transient expression of transcription factors.**

Sameehan Mahajani^1,2*^, Anupam Raina^1,2^, Claudia Fokken^1^, Sebastian Kügler^1,2*§^ and Mathias Bähr^1,2§^.

^1^Department of Neurology, University Medical Center Göttingen, Göttingen, Germany. ^2^Center for Nanoscale Microscopy and Molecular Physiology of the Brain at Department of Neurology, University Medical Center Göttingen, Göttingen, Germany.

§ co-last authors

*Correspondence:

Dr. Sebastian Kügler ([Sebastian.kuegler@med.uni-goettingen.de](mailto:Sebastian.kuegler@med.uni-goettingen.de))

Dr. Sameehan Mahajani ([Sameehan.mahajani@med.uni-goettingen.de](mailto:Sameehan.mahajani@med.uni-goettingen.de))

**Supplementary figure legends for Figures 1-6**

**Supplementary Figure 1. Schematics of Adeno-associated viral vectors.**

(A-B) Representative diagrams of adeno-associated viral vectors encoding the three different rat transcription factors namely, rLmx1a, rNurr1 and rPitx3, used for patterning hiPSCs to dopaminergic neurons (A) along with AAV6-HBA-EGFP, used as a control. Adeno-associated viral vectors encoding α- and γ-synuclein with EGFP as a reporter along with AAV-hSyn1-EGFP, used for studying Synuclein toxicity in hiPSCs derived neurons (B). ITR, inverted terminal repeat; WPRE, woodchuck hepatitis virus post-transcriptional control element; BGH, bovine growth hormone polyadenylation site; Int-a, intron; SV40, simian virus 40; TB, transcription blocker; AU1, AU1 epitope tag; EGFP, enhanced green fluorescent protein; HBA, human beta-actin CMV fusion promoter; hSyn1, human synapsin-1 gene promoter.

**Supplementary Figure 2. Transduction efficiency of Lentiviral and Adeno-associated viral vectors in hiPSCs.**

(A) Representative live cell images of CT-01 hiPSCs transduced with pLV-hUbC-EGFP at different viral titres ranging from 10^8^ to 10^3^ per well along with an uninfected control. Images were recorded three days after transduction (3 DPT). (B) Quantitative analysis of EGFP^+^ cells normalized to total number of cells. Scale bar: 100µm. In all graphs, bars represent the average percentage ± SD from three independent experiments and independent transductions for each viral titre. *p<0.05; **p<0.01; ***p<0.001; ****p<0.0001, one-way ANOVA followed by Bonferroni post hoc test. (C) Representative live cell images of hiPSCs transduced with AAV6-HBA-EGFP at different viral titres ranging from 10^8^ to 10^3^ per well along with an uninfected control. Images were recorded three days after transduction (3 DPT). (D) Quantitative analysis of EGFP^+^ cells normalized to total number of cells. Scale bar: 100µm. In all graphs, bars represent the average percentage ± SD from three independent experiments and independent transductions for each viral titre. **p<0.01; ***p<0.001; ****p<0.0001, one-way ANOVA followed by Bonferroni post hoc test.

**Supplementary Figure 3. Detection of Adeno-associated viral vector mediated overexpression of transcription factors.**

(A) Experimental design to determine the overexpression of transcription factors along with EGFP as a control. CT-01 hiPSCs was transduced with AAV-HBA-rLmx1a-AU1, AAV-HBA-rNurr1 or AAV-HBA-Pitx3-AU1 and lysates were collected at DPT 5 and DPT 10. (B) Quantitative analysis of mRNA expression of EGFP in hiPSCs transduced with AAV-HBA-EGFP at different time points. (C-D) Quantitative analysis of mRNA expression of endogenous hNurr1 (C) or hPitx3 (D) in hiPSCs after transducing them with AAV-HBA-rLmx1a-AU1 and AAV-HBA-rPitx3-AU1 (C) or with AAV-HBA-rLmx1a-AU1 and AAV-HBA-rNurr1 (D) respectively. (E) Quantitative analysis of mRNA expression of endogenous hLmx1a in CT-01 or PD-02 hiPSCs during pharmacological compounds patterning at DIV 5 and DIV 10. In all graphs, data represented as fold change (± SD) as compared to their respective untreated control at the respective time point from three independent experiments. (F) Representative Western blot images of protein lysates collected at DPT 5 and DPT 10 with or without the rTFs viral vector. Respective lysates from rat cortical neurons were used as controls and equal loading of sample in each well was ensured with controls Actin and MemCode.

**Supplementary Figure 4. Quantification of percentage of neurons and dopaminergic neurons obtained after using combination of transcription factors or with the addition of pharmacological compounds.**

(A-B) Experimental representation for determining the number of dopaminergic neurons obtained after using either combination of transcription factors (A) or with the addition of pharmacological compounds (B). Confluent CT-01 hiPSCs were transduced with a combination of AAV viral vectors encoding rat transcription factors at DIV 0, plated on coverslips at DIV 15 and analyzed at DIV 20 for neuronal markers (A). On the other hand, CT-01 hiPSCs transduced with AAV viral vectors encoding transcription factors at DIV 0 were cultured in the presence of pharmacological compounds, plated on coverslips at DIV 20 and analyzed at DIV 25 (B). (C) Representative fluorescence images of immunoreactivity for dopaminergic neuronal marker (TH; red) and neuronal marker (β-Tubulin; green) at DIV 20 after using combination of transcription factors. Nuclei are counterstained with DAPI (blue). Scale bars: 100µm. (D) Representative fluorescence images of immunoreactivity for dopaminergic neuronal marker (TH; red) and neuronal marker (β-Tubulin; green) at DIV 25 after using pharmacological compounds along with each of the transcription factors. Nuclei are counterstained with DAPI (blue). Scale bars: 100µm. (E) Quantitative analysis of total percentage of neurons obtained after using combination of transcription factors for patterning. Data represent the average percentage of total neurons out of total number of cells in culture (β-Tubulin^+^/DAPI^+^). (F) Quantitative analysis of total percentage of dopaminergic neurons obtained after using combination of transcription factors for patterning. Data represent the average percentage of total dopaminergic neurons out of the total number of neurons in culture (TH^+^/β-Tubulin^+^). (G) Quantitative analysis of total percentage of neurons obtained after using pharmacological compounds with each of the transcription factors for patterning. Data represent the average percentage of total neurons out of the total number of cells in culture (β-Tubulin^+^/DAPI^+^). (H) Quantitative analysis of total percentage of dopaminergic neurons obtained after using pharmacological compounds with each of the transcription factors for patterning. Data represent the average percentage of total dopaminergic neurons out of the total number of neurons in culture (TH^+^/β-Tubulin^+^). (I) Quantitative analysis of total percentage of neurons obtained after using either transcription factor (Lmx1a), pharmacological compounds or the combination of transcription factor and pharmacological compounds for patterning. Data represent the average percentage of total neurons out of the total number of cells in culture (β-Tubulin^+^/DAPI^+^). (J) Quantitative analysis of total percentage of neurons obtained after using either transcription factor (Lmx1a), pharmacological compounds or the combination of transcription factor and pharmacological compounds for patterning. Data represent the average percentage of total dopaminergic neurons out of the total number of neurons in culture (TH^+^/β-Tubulin^+^). In all graphs, bars represent the average percentage ± SD from three independent differentiations and independent transductions for each condition. *p<0.01; **p<0.001, one-way ANOVA followed by Bonferroni post hoc test.

**Supplementary Figure 5. Generation of hiPSCs derived glutamatergic neurons using pharmacological compounds.**

(A) Schematic representation of generation of CT-01 hiPSCs derived glutamatergic neurons using pharmacological compounds as previously shown by *Vazin et al, 2014.* (B) Representative fluorescence images of immunoreactivity for glutamatergic neuronal marker (CaMKIIβ; red) and neuronal marker (β-Tubulin; green) in hiPSCs-derived glutamatergic neurons at DIV 18. Nuclei are counterstained with DAPI (blue). Scale bars: 100µm. (C) Quantitative analysis of total percentage of neurons (β-Tubulin^+^) and glutamatergic neurons (CaMKIIβ^+^) obtained after pharmacological compounds patterning. Data represent the average percentage ± SD of total neurons (β-Tubulin^+^/DAPI^+^) and total glutamatergic neurons (CaMKIIβ^+^/DAPI^+^) of all cells in culture.

**Supplementary Figure 6. Synuclein overexpression in hiPSCs derived neurons.**

(A) Experimental design to determine overexpression of synucleins in CT-01 hiPSCs derived neurons by Western blot. After using AAV-HBA-rLmx1a-AU1 to pattern CT-01 hiPSCs into dopaminergic neurons, the neurons were transduced with AAV-hSyn1-α-synuclein-EGFP, AAV-hSyn1-γ-synuclein-EGFP or AAV-hSyn1-EGFP and cell lysates were collected at DIV 25 (i.e. DPT 10). (B) Representative Western blot images of protein lysates collected at 10 DPT with α- or γ-synuclein or EGFP viral vector. Data normalized and controlled with the help of Actin and EGFP respectively. (C-D) Representative fluorescence images of immunoreactivity for synucleins (Pan-synuclein; red) and EGFP (green) in CT-01 hiPSCs-derived neurons transduced with either α-synuclein (C) or EGFP (D) at DIV 25 (DPT 10). Nuclei are counterstained with DAPI. Higher magnification fluorescence images shown for α-synuclein transduced neurons in C’ and C’’ and for EGFP transduced neurons in D’ and D’’. White arrow indicates infected EGFP positive neurons whereas white arrowheads indicate uninfected neurons. Scale bars: 100µm (C-D); 10µm (C’, D’, C’’, D’’).
