# Supplementary material for: Homogenous generation of dopaminergic neurons from multiple hiPSC lines by transient expression of transcription factors
Source: Cell Death Dis. 2019 Nov 27;10(12):898. doi: 10.1038/s41419-019-2133-9 (PMC6881336; doi:10.1038/s41419-019-2133-9)
Supplement: Supplementary file 8 — Supplementary Table [file 41419_2019_2133_MOESM8_ESM.docx]

**Homogenous generation of dopaminergic neurons from multiple hiPSCs lines by transient expression of transcription factors.**

Sameehan Mahajani^1,2*^, Anupam Raina^1,2^, Claudia Fokken^1^, Sebastian Kügler^1,2*§^ and Mathias Bähr^1,2§^.

^1^Department of Neurology, University Medical Center Göttingen, Göttingen, Germany. ^2^Center for Nanoscale Microscopy and Molecular Physiology of the Brain at Department of Neurology, University Medical Center Göttingen, Göttingen, Germany.

§ co-last authors

*Correspondence:

Dr. Sebastian Kügler ([Sebastian.kuegler@med.uni-goettingen.de](mailto:Sebastian.kuegler@med.uni-goettingen.de))

Dr. Sameehan Mahajani ([Sameehan.mahajani@med.uni-goettingen.de](mailto:Sameehan.mahajani@med.uni-goettingen.de))

**Table 1**

**Table 1. Primers used for RT-PCR.**

| **Target** | **Forward primer sequence** | **Reverse primer sequence** |
| --- | --- | --- |
| RAT_Lmx1a | GGACTGTCTGTGCGAGTGGT | GTCTCCGAGCCAGCTTCTTC |
| HUMAN_Lmx1a | CAAGAGCGACGACGAGGAAT | GGTCCTTGCCGTCCTCAGC |
| RAT_Nurr1 | AACTACAGCACCGGCTACGA | CTTGATGCTGCTCTGCTGTC |
| HUMAN_Nurr1 | CGCCTTCCTGGAACTGTTCG | CGCCGTTGCAGAAGATCAGT |
| RAT_Pitx3 | GGGAGACACATATCGCTAC | GCGTCAGACAGAGACAGA |
| HUMAN_Pitx3 | GACTGAAGGCCAAGCAGCAC | TTCCACGGCGTACTGACAC |
| EGFP | ATATCATGGCCGACAAGCAG | TGGCGGATCTTGAAGTTCAC |
| Beta-Actin | ACTTGGCCACGGTCTCTTGT | GTTGGCCACGTTGTCCATGA |
| GAPDH | TGAGCAGTCCGGTGTCATAC | CTGAGAGGCGGGAAAGTTG |
